# *Oreocharis flavovirens*, a new species of Gesneriaceae from Southern Gansu Province, China

**DOI:** 10.3897/phytokeys.157.31732

**Published:** 2020-08-26

**Authors:** Wei-Hua Qin, Dong-Dong Ding, Zhong-Lin Li, Yun-Feng Gao, Shu Li, Xin Hong

**Affiliations:** 1 Nanjing Institute of Environmental Sciences, Ministry of Ecology and Environment of the People's Republic of China, CN–210042, Nanjing, Jiangsu Province, China Nanjing Institute of Environmental Sciences, Ministry of Ecology and Environment of the People's Republic of China Nanjing China; 2 School of Resources and Environmental Engineering, Anhui University, CN–230601, Hefei, Anhui Province, China Anhui University Hefei China; 3 Administration of Yuhe Provincial Nature Reserve, CN–746000, Longnan, Gansu Province, China Yuhe Provincial Nature Reserve Longnan China; 4 The Gesneriad Conservation Center of China, Guangxi Key Laboratory of Plant Conservation and Restoration Ecology in Karst Terrain, Guangxi Institute of Botany, Guangxi Zhuang Autonomous Region and Chinese Academy of Sciences, CN-541006, Guilin, Guangxi Zhuang Autonomous Region, China Guangxi Institute of Botany, Guangxi Zhuang Autonomous Region and Chinese Academy of Sciences Guilin China

**Keywords:** Gansu of China, new taxon, *
Oreocharis
*

## Abstract

*Oreocharis flavovirens* is a new species of Gesneriaceae from Gansu, China and is described and illustrated here. It is morphologically similar to *O. glandulosa*, *O. humilis* and *O. farreri*, but those congeners of this new taxon can be distinguished by several salient characters. A description of *O. flavovirens*, together with illustrations and photos, are presented.

## Introduction

In the summer of 2018, two of the authors (QWH and GYF) encountered an unknown Gesneriaceae species with young flowers during a botanical survey in Gansu Province. Subsequently, the plants were monitored in the field and flowering specimens were collected in autumn. The gross morphology, such as leaves in a basal rosette with spiral leaf arrangement, shape of the corolla and pistil, including stigma, indicates that this taxon can be assigned to *Oreocharis* Benth., which now includes species from eleven former genera (Möller et al. 2011, 2014, 2015). Many new taxa of this genus have been discovered and published in recent years (e.g. Cai et al. 2017, Chen et al. 2016, 2017a,b, 2018, Do et al. 2017, Guo et al. 2018, Li et al. 2017, Wei et al. 2016, Yang et al. 2017, Möller et al. 2016, 2018).

After thorough comparisons of diagnostic morphological and anatomical features of similar taxa from China, Vietnam and Thailand (Pan 1987, Wang et al. 1998, Li and Wang 2005, Wei et al. 2010) and herbarium specimens also being consulted, it was concluded that it was a species new to science and thus described and illustrated here.

## Material and methods

Measurements and morphological character assessments of the new species were performed and described, using specimens worked on by the authors. All available specimens of *Oreocharis* stored in the following herbaria in China, Russia, the United States and the United Kingdom were examined (codes according to Thiers 2015+): E, GH, IBK, K, KUN, MO, PE and US. In addition, images of other type specimens were obtained from Tropicos (http://www.tropicos.org) and JSTOR Global Plants (http://plants.jstor.org). All morphological characters were studied under dissecting microscopes and are described using the terminology presented by Wang et al. (1998) and Li and Wang (2005).

## Taxonomic treatment

### Oreocharis flavovirens

Taxon classificationPlantaeLamialesGesneriaceae

Xin Hong sp. nov.

5089CC8E-0CDB-5F20-92CC-67120256040F

urn:lsid:ipni.org:names:77211187-1

Figures 1 , 2

#### Diagnosis.

*Oreocharis flavovirens* can be diagnosed as a new species from all others in the genus by the upturned corolla tube combined with its rare greenish-yellow colour.

#### Type.

CHINA. Gansu Province: Yuhe Provincial Nature Reserve, Longnan City, 33.08426°N, 105.27858°E, 1,193 m a.s.l., 5 September 2018, flowering, Xin Hong: *HX18090510* (holotype: IBK; isotype: PE).

**Figure 1.**
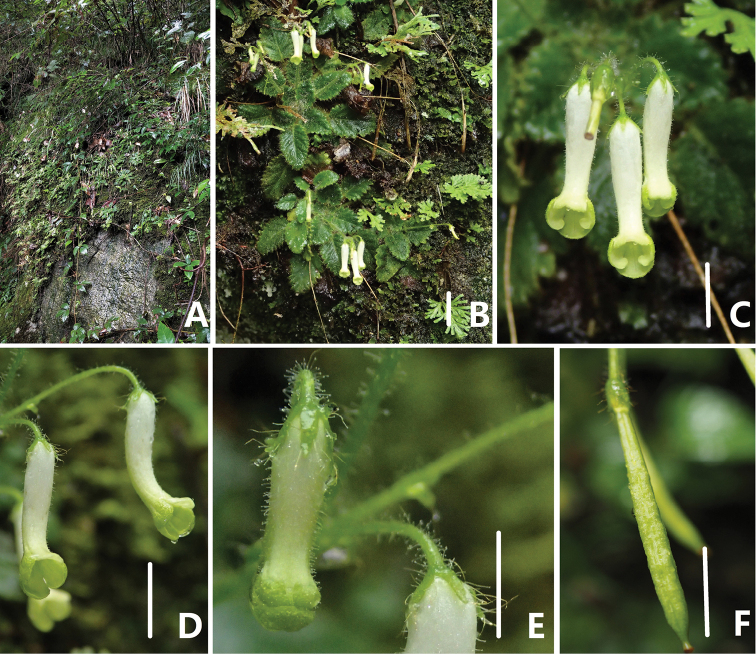
*Oreocharis flavovirens* Xin Hong in natural habitat **A** habitat, growing on the surface of rocks **B** vegetative part of plants **C** top view of corolla **D** lateral view of corolla **E** bud, showing the shape and indumentum of calyx **F** young capsule. Scale bars: 2 cm (**B**); 1 cm (**C–F**).

#### Description.

Perennial, rosette herbs. ***Leaves*** basal, spirally arranged, 4–20, petiolate; petioles terete, 1.4–4 cm long, ca. 2 mm in diameter, densely reddish-brown long woolly and white glandular hairy; leaf blades ovate to obovate or elliptic, 3–6 × 1.5–3 cm, bases cuneate, slightly unequal, margins crenate to lobulate, apices obtuse, papery, adaxially sparsely rust-brown villous hairy, green, abaxially densely brown villous along veins, pale green; midrib usually vivid when fresh, lateral veins 3–5 on each side of midrib, distinct, concave adaxially, prominent abaxially. ***Inflorescences*** cymes, axillary, 1–2(–3)-branched, 1–6(–10)-flowered; peduncles 4–10 cm long, pale green, densely white glandular hairy and sparsely brown pilose; pedicels 1–3(–5) cm long, with indumentum as on the peduncle. ***Bracts*** 2, ca. 3 × 1 mm, lanceolate, margins entire, green, glabrous inside, brown pilose and sparsely glandular hairy outside; bracteoles similar but smaller, ca. 1.5 × 0.5 mm. ***Calyx*** actinomorphic, 5- sect from base, segments oblong to linear-lanceolate, 2–3.5 × ca. 1.0 mm, green, glandular hairy outside and glabrous inside, margins entire, sometimes revolute when flowering. ***Corolla*** zygomorphic, ca. 2 cm long, greenish-yellow to greenish, lobes greenish, becoming white at tube base, outside densely glandular-pubescent, inside glabrous; tube cylindrical, dilated and slightly narrowing gradually ventricose from base to throat and constricted at the throat, ca. 15 mm long, ca. 3 mm in diameter at base and ca. 2 mm in diameter at the throat; limb slightly 2-lipped; adaxial lip rounded, 2.5–3 × ca. 3 mm, emarginate or rarely undivided, shorter than abaxial lip; abaxial lip 3-sect from above middle, lobes obovate to elliptic, apex rounded, central longer than laterals, 3× 4–ca. 3 mm. ***Stamens*** 4, adnate to corolla 1–4 mm above base, included; filaments slender, the long two ca. 8 mm long, the short two ca. 6 mm long, sparsely glandular-pubescent, free, white to greenish; anthers yellow, basifixed, coherent in pairs, thecae divergent at base, oblong, ca. 0.5 mm long, 2-loculed, dehiscing longitudinally from arcuate slits, connective not projecting, glabrous; staminode 1, glabrous, 0.5–1.5 mm long, adnate to 1 mm above corolla tube base. ***Disc*** ring-like, 1–1.5 mm high, glabrous, entire or subentire, greenish-yellow. ***Pistil*** 1–1.2 cm; ovary narrowly oblong, 1-loculed, ca. 1 cm long; placentas 2, parietal, projecting inwards, 2-cleft, style 1–2 mm long, glabrous; stigma orbicular, emarginated, ca. 2 mm in diameter. ***Capsules*** oblong lanceolate to oblanceolate, straight, 2–4 cm long, dehiscing loculicidally to base; valves 2, glabrous. ***Seeds*** unknown.

**Figure 2.**
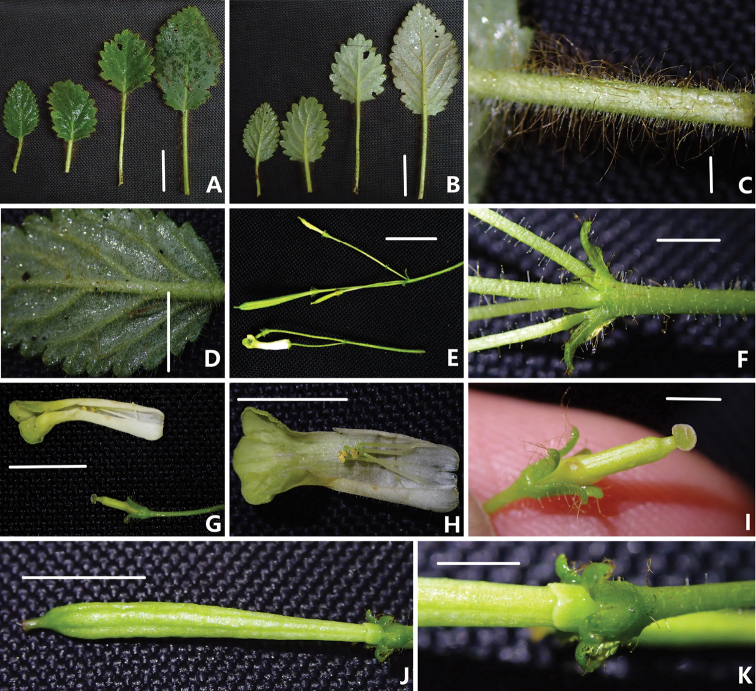
Morphological character of *Oreocharis flavovirens* Xin Hong **A–B** different sizes of leaves (**A** adaxial leaves **B** abaxial leaves) **C** indumentum of petiole **D** abaxial leaf blades, showing veins and indumentum **E** cyme and infructescence **F** bracts **G** dissection of a flower **H** opened corolla, showing stamens and staminodes **I** pistils without corolla, showing stigma **J** young capsule **K** disc and calyx revolute of the young capsule. Scale bars: 2 cm (**A–C**); 1.5 cm (**D**, **E**); 3 mm (**F, I, K**), 1 cm (**G**, **H**, **J**).

#### Etymology.

The specific epithet is derived from its greenish-yellow corolla.

#### Distribution and habitat.

To date, *Oreocharis flavovirens* is only found at the type locality, Yuhe Provincial Nature Reserve, Gansu Province, which is located at the intersection of the Qinling Mountains and the Minshan Mountains. This species grows amongst moss on moist shady surfaces of stones near waterfalls, at an elevation of 950–1200 m a.s.l. The average temperature is 21°C, the average annual precipitation has been calculated as ca. 780 mm. The forest is a subtropical evergreen broad-leaved forest.

#### Notes.

**Figure 3.**
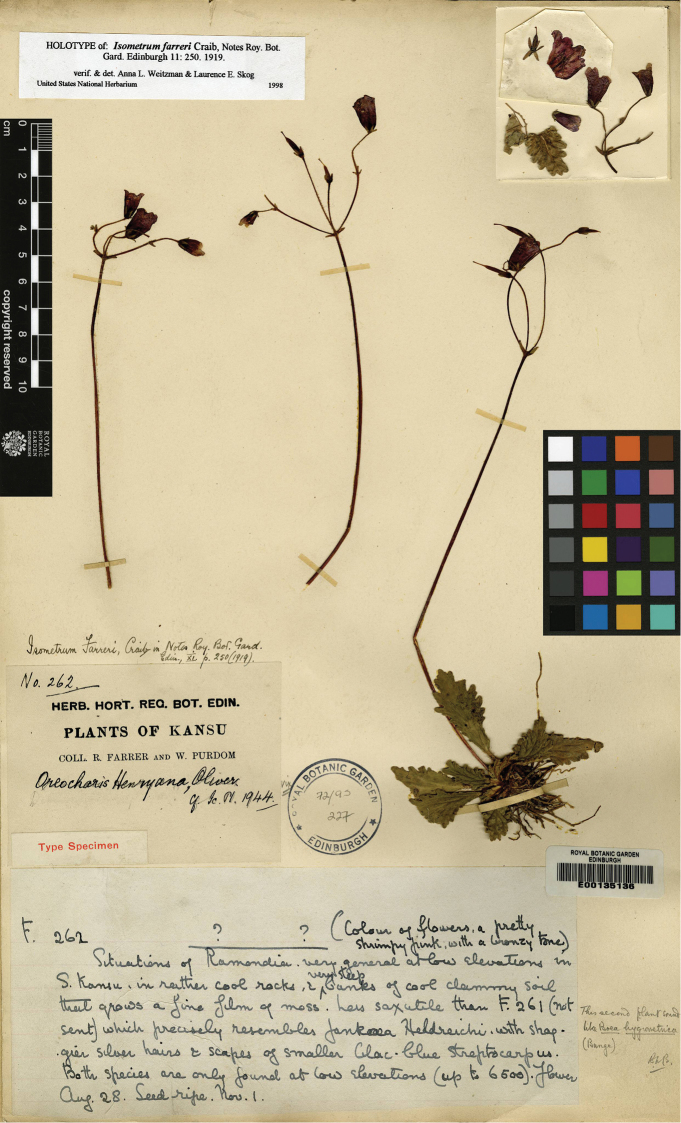
Type of *Oreocharis farreri* (W. G. Craib) M. Möller & A. Weber, stored in Herbarium of Royal Botanic Garden Edinburgh, No. E 00135136.

**Figure 4.**
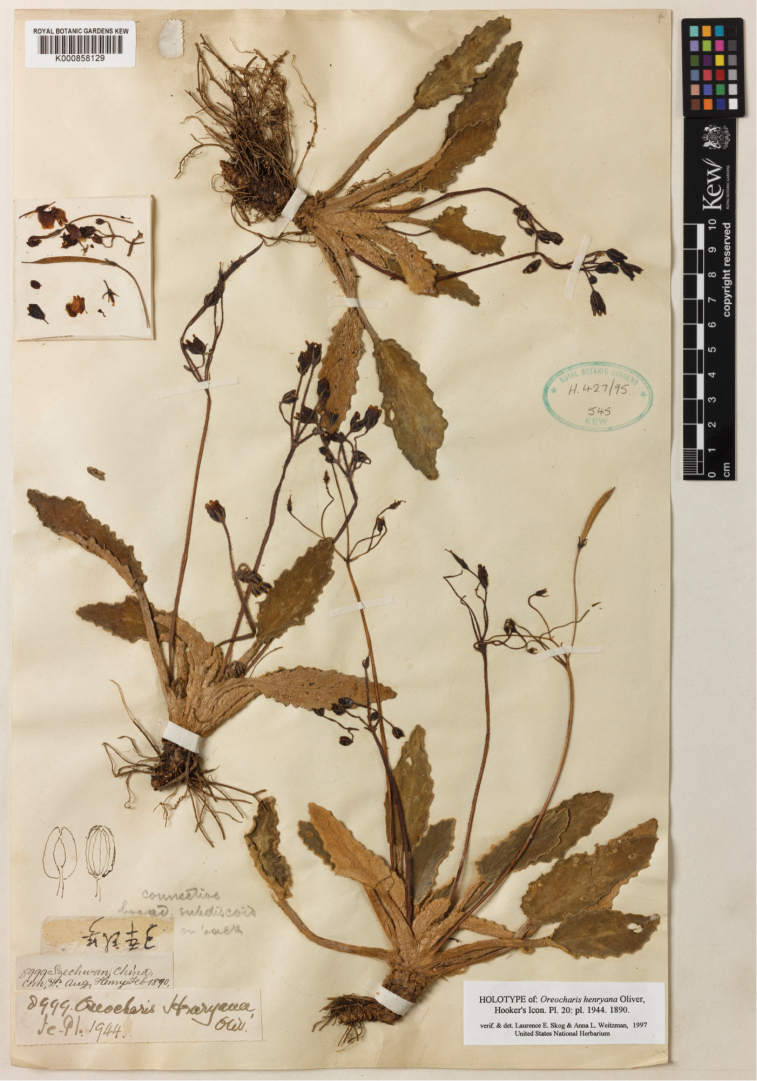
Type of *O. glandulosa* (Batalin) M. Möller & A. Weber, stored in Herbarium of Komarov Botanical Institute, No. LE 01043081.

**Figure 5.**
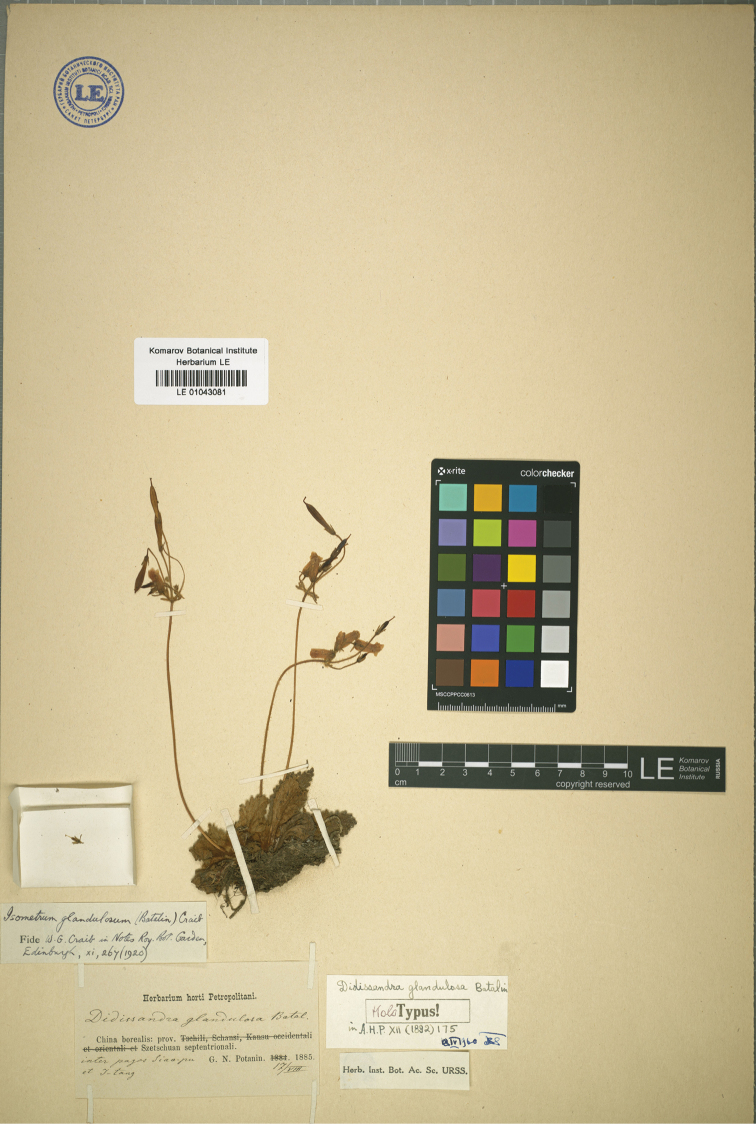
Type of *Oreocharis henryana* Oliv. stored in Herbarium of Royal Botanic Gardens, No. K 000858129.

As is known, *Oreocharis* Benth. is a genus (more than 120 species) in the angiosperm family Gesneriaceae, which are mainly distributed in southern and south-western China, at the same time with a few species extending into Vietnam, Myanmar, India, Bhutan, Japan and Thailand (Cai et al. 2017, Möller et al. 2016, 2018, Xu et al. 2017). SW China is rich in species diversity of the genus in China, especially on the north-facing shady slope nearby the summit of southern Yunnan Province and most species occur in relatively restricted and geographically isolated localities with very few widely distributed (Li and Wang 2005, Wei et al. 2010, Möller et al. 2011). Only three species of this genus were found in S. Gansu province before 2019, viz. *Oreocharis farreri* (W. G. Craib) M. Möller & A. Weber, *O. glandulosa* (Batalin) M. Möller & A. Weber and *O. henryana* Oliv. *O. farreri* was first published as *Isometrum farreri* base on the type specimens: *Farrer et Purdom 262* [E, barcode no. 00135136, Fig. 3], which grows at low elevations on rather cool rocks or very steep banks of cool clammy soil that grows a fine film of moss in S. Gansu Province (Craib 1920). *O. glandulosa* was first described as *Didissandra glandulosa* by A.T. Batalin in 1892, based on the specimens [LE, barcode no. 01043081, Fig. 4] from G.N. Potanin’s trip from 1884 to 1886, collected on the way from Songpan County, Tibetan Qiang Autonomous Prefecture of Ngawa, NE Sichang Province to Wenxian County, Longnan City, S. Gansu Province on 17 August 1885 (Batalin 1892, Bretschneider 1898). *O. henryana* was described and illustrated, based on the type specimens: *A. Henry 8999* [K, barcode no. 000858129, Fig. 5], growing on shady and damp rocks in montane regions of Sichuan Province (Hooker 1890). No new species of *Oreocharis* were described from between the early 19^th^ and late 20^th^ Century in the regions, the new findings complementing the species richness of the genus in Central China. Due to the high endemism in the genus (Chen et al. 2017b, 2018), Table 1 details the differences between these species growing in the same regions.

Obviously, the genus is special for its remarkable floral diversity and it has made this genus to be one of the most taxonomy-difficult groups in the family. The new species, *Oreocharis flavovirens* which has a light-yellow cylindrical corolla with a distinct upturned tube, is a good example. The shape of the corolla tube, cylindrical and upturned, is a distinct character that not many species in *Oreocharis* possess. If we only consider the similarity of corolla tube shape, it is close to *Oreocharis tubiflora* K.Y. Pan and *O. argyreia* Chun ex K.Y. Pan, including constriction at the mouth but the latter two are lacking the upturned corolla tube. Given the corolla shape, the corolla of several species of former *Ancylostemon* Craib are similar too, except the tubes are straight or slightly turned down, but not up, though the flowers are predominantly yellow (rarely pink in *A. ronganensis* K. Y. Pan=*Oreocharis ronganensis* (K.Y.Pan) Mich.Möller & A.Weber), but not greenish-yellow. On the other hand, the upturned tube is more reminiscent of former *Opithandra wentsaii* Z.Yu Li (=*Oreocharis wentsaii* (Z. Yu Li) M. Möller & A. Weber) and former *Opithandra pumila* (W.T.Wang) Wang (=*Oreocharis pumila* (W.T.Wang) Mich.Möller & A.Weber), only here the tubes of previous *Opithandra* are slightly more trumpet-shaped in dark pink or pink and have two fertile stamens rather than four (Wang et al. 1998, Li and Wang 2005, Wei et al. 2010). All in all, the upturned corolla tube combined with its greenish-yellow colour could be used alone to differentiate the new species from others in the genus.

Furthermore, although the genus *Oreocharis* was redefined to accommodate species with distinctive floral morphologies from ten other genera, based on molecular phylogenetic studies in the last two decades, the evolutionary trends of the floral characters have not yet been understood comprehensively. The major causes of the incongruence and conflict between classical taxonomy and molecular phylogenetic studies for *Oreochariss.l.* remain largely unexplored. There are other similar examples in Gesneriaceae of Asian, for example, *Primulinas.l.* (Wang et al. 2011, Weber et al. 2011a) and *Petrocodons.l.* (Weber et al. 2011b, Lu et al. 2017).

#### Additional specimens examined (paratypes).

Gansu Province: Yuhe Provincial Nature Reserve, Longnan City, 24 September 2019, in fruit, Yun-Feng Gao et al.: *WF19092401* (AHU).

**Table 1.** Diagnostic character differences amongst *Oreocharis flavovirens* sp. nov., *O. glandulosa*, *O. humilis* and *O. farreri*.

**Characters**	***Oreocharis viridifrons***	***O. glandulosa***	***O. humilis***	***O. farreri***
Shape of leaf blade	ovate to obovate or elliptic	lanceolate-ovate	elliptic to lanceolate	rhombic-ovate to obovate or elliptic
Indumentum of leaf blade	adaxially sparsely rust-brown villous hairy	adaxially densely brownish villous	adaxially sparsely brown villous, glabrescent	gray pubescent
Number of lateral veins on each side of midrib	3–4	5–6	3–5	4–6
Size of Bracts	3 mm	5 mm	2-4 mm	3.5–5 mm
Shape of tube	campanulate-tubular, laterally compressed at mouth	tubular to subcampanulate	tubular	campanulate-tubular
Size of corolla	ca. 20 mm long	10–15 mm long	11–15 mm long	9–11 mm long
Color of corolla	greenish-yellow to greenish	pale purple	yellow-white	purple-pink to orange-pink
Shape and size of adaxial lip	emarginate or rarely undivided, 2.5-3 mm	emarginate or rarely undivided, 4 mm	2-lobed, 2 mm	emarginate
size of abaxial lip	3–4 mm, longer than to nearly equalling abaxial lip	2 mm, shorter than to nearly equalling abaxial lip	3.5 mm, longer than to nearly equalling abaxial lip	2 mm, shorter than abaxial lip
Staminodes	adnate to 1 mm above corolla tube base	adnate to 0.5 mm above corolla tube base	adnate to 3.5 mm above corolla tube base	adnate to 1 mm above corolla tube base
Ovary	10 mm	3-7 mm	6–8 mm	4 mm
Stigma	peltate, orbicular	emarginate	2-lobed	oblate

## Supplementary Material

XML Treatment for Oreocharis flavovirens
